# Anti-synthetase syndrome: a rare and challenging diagnosis for bilateral ground-glass opacities—a case report with literature review

**DOI:** 10.1186/s12890-020-01388-0

**Published:** 2021-01-06

**Authors:** Nasam Alfraji, Usman Mazahir, Moiuz Chaudhri, Jeffrey Miskoff

**Affiliations:** 1 Department of Internal Medicine, Jersey Shore University Medical Center, Hackensack Meridian Health, Neptune, NJ 07753 USA; 2 Department of Pulmonology and Critical Care, Jersey Shore University Medical Center, Neptune, NJ 07753 USA

**Keywords:** Anti-synthetase syndrome, Autoimmune disease, Interstitial lung disease, Corticosteroids

## Abstract

**Background:**

Anti-synthetase syndrome (ASS) is an uncommon immune-mediated entity characterized by myositis, interstitial lung disease (ILD), non-erosive arthritis, and less common features such as fever, Raynaud’s phenomenon, and skin changes in association with anti-aminoacyl-transfer-RNA antibodies, most commonly anti-Jo-1 antibodies.

**Case presentation:**

We present a challenging and rare case of ASS-associated ILD presenting with unexplained respiratory symptoms and bilateral infiltrates on chest imaging during the COVID-19 pandemic. High clinical suspicion for ASS with early appropriate therapy with corticosteroids and immunosuppressive agents led to marked clinical improvement.

**Conclusion:**

High index of suspicion for ASS is mandated in patients with unexplained ILD. A comprehensive autoimmune work-up is important as an early treatment with corticosteroids with or without immunomodulators improves patient outcomes and survival in an otherwise poor prognostic disease.

## Background

Anti-synthetase syndrome (ASS) is a rare multisystemic autoimmune disease with variable manifestations ranging from myositis, interstitial lung disease (ILD), and non-erosive arthritis to less common features such as fever, Raynaud ‘s phenomenon, and skin changes in the setting of detectable anti-aminoacyl-transfer-RNA antibodies, most commonly anti-Jo-1 antibodies [1].

ASS has unclear pathogenesis; however, it is hypothesized that immune intolerance and immune self-reactivity have major implications [2, 3]. It is thought to develop in genetically susceptible individuals in the setting of triggering environmental factors and immune system activation [2, 4].

ASS is considered by some authors as a separate disease entity within the idiopathic inflammatory myopathy (IIM) besides dermatomyositis (DM) and polymyositis (PM) [2]. ASS occurs mainly in adults with average age 50 years and more commonly in Caucasian females than males [5, 6].

The prevalence of ILD has been reported in 69–100% of ASS patients in several studies which is also the major predictor of mortality and morbidity. ILD is the presenting feature of ASS in only 15–30%. Patients usually present with exertional dyspnea with or without dry cough. Pulmonary hypertension also reported with or without concomitant ILD [2, 6].

ASS is considered a challenging and under-recognized clinical entity that requires a high index of suspicion as it can mimic other diseases such as infections especially if presenting with incomplete disease pattern [7]. Early recognition, monitoring for new disease manifestations, and treatment with corticosteroids and other immunosuppressive agents result in a better outcome and prognosis [7].

We inhere report a rare case of ASS with a primary and an isolated ILD, which presented during the COVID-19 pandemic era rendering the diagnosis more challenging. This case has been reported in line with the CARE criteria.

## Case presentation

A 51-year-old white female who was referred from our outpatient pulmonary clinic to the ED (emergency department) for worsening respiratory distress and six weeks history of persistent dry cough. Prior to this admission, the patient was seen at an urgent care and was prescribed a 10 days course of oral antibiotic empirically without significant improvement. Subsequently, the patient was seen in our outpatient pulmonary clinic for a working diagnosis of pneumonia and a previous chest x-ray depicting bibasilar opacities. In the office, the patient complained of dyspnea with ambulation. She was tested negative for COVID-19. Therefore, patient was prescribed oral doxycycline 100 mg twice daily empirically for 14 days and 40 mg of oral prednisone for 5 days. The patient was advised to communicate her progress with the treating physician, so her treatment protocol can be adjusted if needed. However, attempts to wean her prednisone made her dyspnea worse with pulse oximetry reportedly in high 80 s% on ambulation. PFTs (pulmonary function tests) outpatient showed restrictive lung pattern with reduced FEV-1 (forced expiratory volume in 1 s) at 52%, and reduced FVC (Forced vital capacity) at 47%.

Patient reported having associated generalized fatigue, but no weight loss. She denied any hemoptysis, chest pain, fever, chills, night sweats, epistaxis, dry eyes, dry mouth, vision changes, photosensitivity, oral ulcer, dysphagia, abdominal pain, nausea, vomiting, constipation, or diarrhea. She denied any urinary disturbances, myalgia, joint pain or swelling, blood in urine or stool, or any Raynaud's type symptoms. Patient reported a recent travel history to Florida, but no history of sick contacts. She endorsed a family history only significant for Crohn's disease in father and daughter. She reported no alcohol use and no smoking history. The patient did not have any occupational or pet exposure.

In the ED, the patients' vital signs were a temperature of 97.6 F, respiratory rate of 26 per minute, oxygen saturation of 88–90% on room air which improved to 94% with 3 L of oxygen on nasal cannula, and blood pressure of 102/53 mmHg. Patient’s body weight was 75.3 kg and her height was 157 cm. On physical exam, patient noticed to be dyspneic and tachypneic. She was alert and oriented to time, place and person. Head and neck examinations were unremarkable for lymphadenopathy, jugular venous distention (JVD), nasal/oral ulcerations, or any other lesions. On chest auscultation, reduced breath sounds were evident at the lower lung fields. Cardiology, gastrointestinal, and neurological examinations were unremarkable. No joint tenderness/swelling or muscle tenderness/weakness appreciated at the musculoskeletal examination. Skin examination revealed no rash or other lesions. The patient was admitted for further evaluation, beginning with retesting for COVID-19 and additional workup.

Initial complete blood count, renal, and liver panel were all within normal limits except for normocytic anemia (Table 1). Erythrocyte sedimentation rate (ESR) was 49 mm/h (0–15 mm/h), C-reactive protein (CRP) was 3.24 mg/dL (0.00–0.74 mg/dL), and anti-nuclear antibodies 2.09 (0.0–0.90). Urinalysis and complement levels were unremarkable. Creatine kinase (CK) and aldolase were 326 iu/L (22–232 iu/L), 39.5 u/L (1.5–8.1 u/L) respectively. The COVID-19 RT-PCR (Reverse transcription polymerase chain reaction) testing was negative twice as an inpatient.

**Table 1:** Summary of main laboratory investigations at admission and follow-up

Lab	Admission	3-months follow-up	Reference value
Hemoglobin	9.8	12.7	12–16 g/dL
WBC	10.3	6.2	4.5–11.0 K/uL
Platelets	285	325	140–450 K/uL
BUN	4	–	5–25 mg/dL
Creatinine	0.32	–	0.61–1.24 mg/dL
Glomerular filtration rate	> 60	–	> 60
ALT	46	14	10–60 U/L
AST	57	16	10–42 U/L
ESR	49	22	0–15 mm/h
CRP	3.24	0.45	0.00–0.74 mg/dL
Rheumatoid factor	< 20	–	< 20 U/mL
Anti-nuclear antibodies	2.09	–	0.0–0.90
C3	106	–	85–170 mg/dL
C4	22.2	–	16–40 mg/dL
JO-1 antibody	191	–	0–40 AU/mL
c-ANCA	< 1.20	–	< 1.20
p-ANCA	0.0	–	0–19 U/mL
Anti-dsDNA	< 1:10	–	< 1:10
Scleroderma (scl-70) antibody	3.0	–	0–40 AU/mL
Anti-Smith antibody	1.0	–	0–40 AU/mL
Anti-Sjögren's-syndrome-related Ag A	5.0	–	0–40 AU/mL
Anti-Sjögren's-syndrome-related Ag B	1.0	–	0–40 AU/mL
Anti-RNP antibody	8.0	–	0–40 AU/mL
Anti-cyclic citrullinated antibodies	3.0	–	0–19 units
Legionella antigen, urine	Negative	–	Negative
Streptococcus pneumonia antigen	Negative	–	Negative
Beta-d-glucan	Negative	–	Negative
Procalcitonin	0.11	–	< 0.50 ng/mL
Aldolase	39.5	4.5	1.5–8.1 u/L
Creatine kinase	326	58	22–232 iu/L

**Fig. 1:**
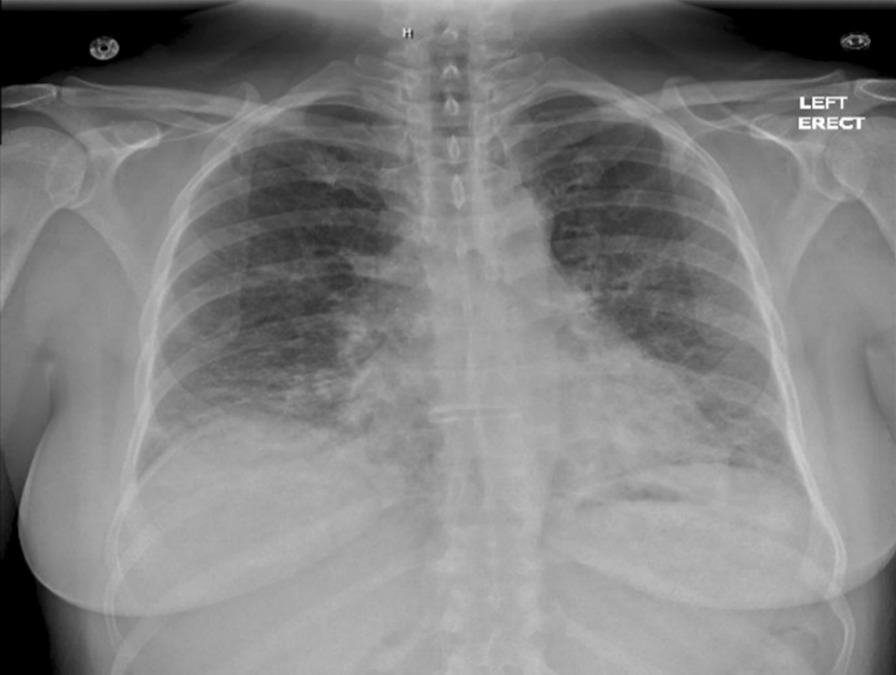
Chest x ray revealing persistent bibasilar infiltrates

**Fig. 2:**
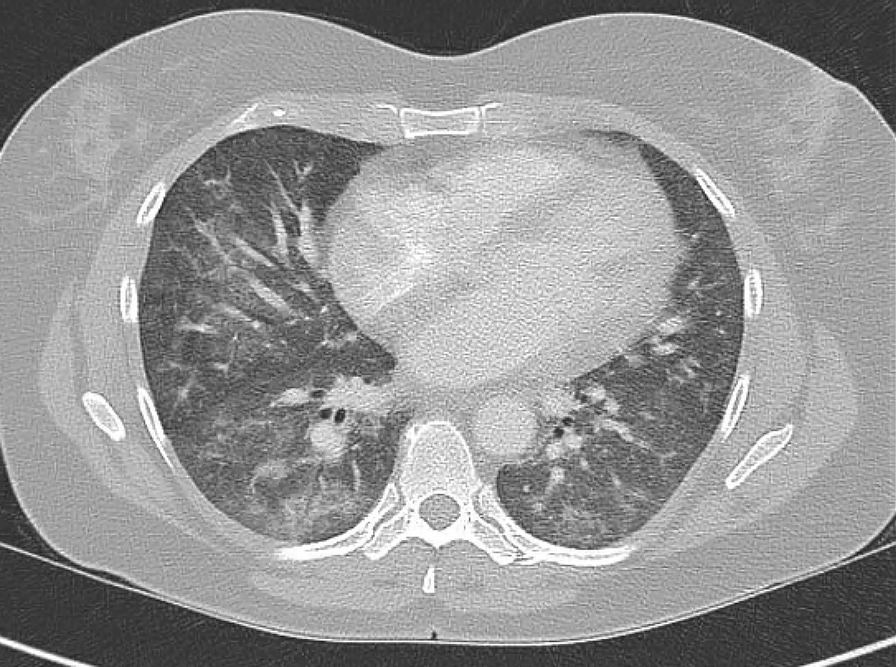
Computed tomography angiography of the chest revealing bilateral ground glass opacities, with no filling defects suggesting pulmonary emboli

A chest x-ray (Fig. 1) showed persistent bibasilar infiltrates, finding similar to previous imaging. A Computed tomography angiography of the chest (Fig. 2) showed bilateral ground glass opacities, with shotty mediastinal lymph nodes, and no filling defects to suggest a pulmonary embolism. Echocardiogram showed normal left ventricular (LV) function, right ventricular function, and pulmonary pressures.

The patient underwent bronchoscopy with right lower lobe transbronchial biopsies which showed mild lung parenchymal inflammation, fibrosis, and reactive epithelial changes without any sign of malignancy. Gomori methenamine-silver (GMS) nitrate stain and acid-fast stains (also known as the Ziehl–Neelsen stain) were negative for *Pneumocystis jiroveci* and Mycobacterium species, respectively. Bacterial and fungal cultures from right lower lobe bronchoalveolar lavage remained negative.

Subsequent autoimmune screening returned strongly positive for anti‑Jo‑1 antibody 191 au/mL (reference range 0–40). Other markers, including rheumatoid factor, anti‑cyclic citrullinated peptide antibodies, anti‑Ro/SSA, and antineutrophil cytoplasmic antibodies were negative.

Therefore, ASS-associated ILD was considered in the setting of clinical and radiographic findings of nonspecific interstitial pneumonia (NSIP) associated with positive anti-Jo-1 antibody.

Patient was started on intravenous methylprednisolone 40 mg every 12 h which failed to improve patient’s symptoms; therefore, she was given intravenous pulse methylprednisolone 1000 mg daily for 3 days. Patient reported some improvement of her symptoms after pulse steroids. She was discharged with 2 L home oxygen as needed and on high dose oral corticosteroids, prednisone 60 mg oral daily. She followed up with rheumatology outpatient two weeks after discharge and was started on oral mycophenolate 500 mg twice daily. However, tapering steroids was difficult and mycophenolate was titrated up to 1500 mg twice daily.

At her 3 months follow-up, the patient continued to have a gradual improvement of her symptoms and she was weaned off oxygen. Chest high-resolution computed tomography (HRCT) at that time showed 20% interval improvement particularly in the lower lobes with improvement of her laboratory markers such as ESR, CRP, CK, and aldolase demonstrated in (Table 1, column 2). Favorably, her prednisone was tapered over six months to 10 mg daily while being on the same dose of mycophenolate.

## Discussion

Anti-synthetase syndrome (ASS) is a rare autoimmune disease characterized mainly by interstitial lung disease (ILD), myositis, and arthritis reportedly in 90% of cases [2, 8]. However, other manifestations like fever, rashes, and Raynaud’s syndrome have also been seen less commonly [2, 8]. ILD was noted to be the initial presentation in 15–30% of ASS patients in various studies [2]. The main serological hallmark of this syndrome is the presence of various autoantibodies to aminoacyl transfer RNA (tRNA) synthetase including Jo-1 and others [2]. ASS was first identified as a clinical entity by Marguerie et al*.* in 1990 [6].

The complete form of ASS includes the triad of ILD, myositis, and arthritis which is only reported in 19.5% at disease onset in Cavagna et al. study and lacking any of these features is considered as an incomplete form of ASS [9]. Patient with incomplete picture might eventually exhibit other manifestations to have a complete form over a variable period of time [9].

In 2016, Trallero‐Araguás et al. studied the clinical manifestations and long-term outcome of 148 patients with anti-Jo-1 syndrome from 18 Spanish hospitals. Most of the cases had an incomplete picture of Anti-synthetase syndrome at onset as follows: isolated ILD 47 (32.4%) patients, isolated myositis 39 (26.9%) patients, and isolated polyarthritis 26 (17.9%) patients. Only a minority had stable disease at the end of follow-up with isolated ILD still reported in 21 (14.5%) of patients, isolated myositis in 23 (15.9%) and isolated polyarthritis in three (2.1%) patients [10]. Mortality rate was reportedly four-fold higher than general population [10].

There are still no unified internationally-recognized ASS classification criteria; however, there are two main classification criteria for ASS, Connors et al. 2010, and Solomon et al. 2011, and both require serological and specific clinical features [2]. Our case was diagnosed in accordance with Connors et al. 2010 criteria that require the presence of anti-synthetase antibodies with one or more of the following: myositis by Bohan and Peter criteria, ILD not explained by other causes, arthritis, persistent fever, Raynaud’s phenomenon, and mechanic's hands [2].

ILD is the main pulmonary manifestation of ASS and the major cause of morbidity and mortality [2]. Patients usually present with dyspnea on exertion accompanied by dry cough [6]. Instrumentally, ILD is defined by PFTs (pulmonary function tests) which show restrictive picture, indicated by total lung capacity (TLC) < 80% predicted, diffusion capacity of lung for carbon monoxide (DLCO) < 70% of predicted value, and/or forced vital capacity (FVC) < 80% predicted, and/ or specific findings on HRCT scans of the lungs [2, 5]. The usual type of ILD seen on HRCT is nonspecific interstitial pneumonia (NSIP) which characterized by ground-glass opacities. Other types include COP (cryptogenic organizing pneumonia) characterized by consolidation and linear opacities, and usual interstitial pneumonia (UIP) characterized by honeycomb pattern and traction bronchiectasis [5, 11]. Bronchoscopy and lung biopsy are not indicated routinely for diagnosis of ASS; however, it could be considered to rule out other causes or exclude infection [2].

There is still no standardized regimen for ASS treatment; however, treatment with prednisone 1 mg/kg/day (or methylprednisolone 1 g IV daily for 3 days, in severe cases) is the most common initial therapy [2]. Additionally, a steroid‑sparing agent (usually mycophenolate or azathioprine) can be used along with steroid and has showed a better survival and fewer relapses in comparison to prednisone monotherapy [2]. Other case studies reported using cyclophosphamide, cyclosporine, and methotrexate, and intravenous immunoglobulins as immunomodulating agents [1, 5, 7]. Rituximab has been used for patients with refractory ILD [8].

Several prognostic factors of poor outcome and reduced survival have been identified in ASS including old age, African-American ethnicity, Male gender, UIP picture on HRCT chest, symptomatic ILD, steroid-resistant ILD, lower numbers of FVC and DLCO at time of diagnosis [1, 7, 8]. However, the severity of ILD remains the ultimate prognostic factor of ASS [1, 5].

The association between ASS and malignancy is controversial. Although some studies including one large cohort study reported no increase in malignancy rates in ASS patients comparing to the general population, other case series and reports mentioned development of different malignancies within variable period of ASS diagnosis [2, 5, 12]. In one of the case series, malignancy involved the breast, stomach, cervix, thyroid, nasopharynx, and salivary duct developed in 8 (6.5%) of 124 patients [12]. Therefore, authors recommend age-appropriate malignancy screening for all patients with ASS [2, 5, 12].

Our unique case was a rare clinical entity which posed a diagnostic challenge as patient presented with worsening dyspnea and dry cough at the onset of COVID-19 pandemic. However, carrying a high index of suspicion and performing a comprehensive work-up led to an early diagnosis of ASS and prompt treatment resulting in a favorable outcome. Our patient showed a fair improvement after intravenous pulse steroids dosing and was started on high dose oral prednisone. Our decision to add a second immunosuppressive agent was based on our patient’s progressive presentation with respiratory symptoms and exertional hypoxia along with the presence of anti-synthetase antibodies. Furthermore, a steroid‑sparing agent (mycophenolate in our case) has been chosen as it showed fewer relapses in comparison to prednisone monotherapy in several studies [2].

## Conclusion

ASS is a rare clinical syndrome with a variable symptomology and combination. Our case emphasizes the importance of considering ASS-associated ILD in our differentials in case of unexplained respiratory symptoms with bilateral grand-glass opacities on chest imaging after excluding other possibilities including infections as early diagnosis and timely treatment would improve patient outcome and survival. Although our case had clinical improvement with corticosteroids treatment and subsequently immunosuppressive agent, there are still no enough standardized guidelines about the management of ASS-associated ILD. Therefore, it requires more randomized controlled trials and research with larger sample size to mainstream the approach for diagnosing and treating ASS.

## Data Availability

Data sharing is not applicable to this article as no datasets were generated or analyzed during the current study.

## Abbreviations

ASS: Anti-synthetase syndrome
ILD: Interstitial lung disease
IIM: Idiopathic inflammatory myopathy
DM: Dermatomyositis
PM: Polymyositis
PFTs: Pulmonary function tests
JVD: Jugular venous distention
ESR: Erythrocyte sedimentation rate
CRP: C-reactive protein
CK: Creatine kinase
RT-PCR: Reverse transcription polymerase chain reaction
TLC: Total lung capacity
LV: Left ventricular
GMS: Gomori methenamine-silver
NSIP: Nonspecific interstitial pneumonia
DLCO: Diffusion capacity of lung for carbon monoxide
COP: Cryptogenic organizing pneumonia
UIP: Usual interstitial pneumonia
HRCT: High-resolution computed tomography
